# Deciphering functional diversification within the lichen microbiota by meta-omics

**DOI:** 10.1186/s40168-017-0303-5

**Published:** 2017-07-19

**Authors:** Tomislav Cernava, Armin Erlacher, Ines Aline Aschenbrenner, Lisa Krug, Christian Lassek, Katharina Riedel, Martin Grube, Gabriele Berg

**Affiliations:** 1 Institute of Environmental Biotechnology, Graz University of Technology, Petersgasse 12, 8010 Graz, Austria; 2 Austrian Centre of Industrial Biotechnolgy GmbH, Petersgasse 14, 8010 Graz, Austria; 3 Institute of Microbiology, Ernst-Moritz-Arndt University of Greifswald, Friedrich-Ludwig-Jahn Strasse 15, 17489 Greifswald, Germany; 4 Institute of Plant Sciences, University of Graz, Holteigasse 6, 8010 Graz, Austria

**Keywords:** Metagenomics, Metaproteomics, Metatranscriptomics, Amplicon sequencing, Lichen symbiosis, *Lobaria pulmonaria*

## Abstract

**Background:**

Recent evidence of specific bacterial communities extended the traditional concept of fungal-algal lichen symbioses by a further organismal kingdom. Although functional roles were already assigned to dominant members of the highly diversified microbiota, a substantial fraction of the ubiquitous colonizers remained unexplored. We employed a multi-omics approach to further characterize functional guilds in an unconventional model system.

**Results:**

The general community structure of the lichen-associated microbiota was shown to be highly similar irrespective of the employed omics approach. Five highly abundant bacterial orders—*Sphingomonadales*, *Rhodospirillales*, *Myxococcales*, *Chthoniobacterales*, and *Sphingobacteriales—*harbor functions that are of substantial importance for the holobiome. Identified functions range from the provision of vitamins and cofactors to the degradation of phenolic compounds like phenylpropanoid, xylenols, and cresols.

**Conclusions:**

Functions that facilitate the persistence of *Lobaria pulmonaria* under unfavorable conditions were present in previously overlooked fractions of the microbiota. So far, unrecognized groups like *Chthoniobacterales* (*Verrucomicrobia*) emerged as functional protectors in the lichen microbiome. By combining multi-omics and imaging techniques, we highlight previously overlooked participants in the complex microenvironment of the lichens.

**Electronic supplementary material:**

The online version of this article (doi:10.1186/s40168-017-0303-5) contains supplementary material, which is available to authorized users.

## Background

Lichens were among the first life forms to conquer life on land already in the Lower Devonian [35]. The drought-tolerant symbiosis evolved an intricate symbiotic architecture, also known as the lichen thallus, which comprises peripheric fungal structures to shelter photosynthetic partners. The long-living lichen thalli also provide microhabitats for diverse microorganisms, in particular fungi and bacteria [5, 27, 28, 31–33, 46, 65]. This new knowledge suggested the emendation of the classic concept of lichens as a bipartite partnership of fungi and algae, especially if the additional partnerships contribute to the global functioning of the symbiosis. First evidence in this direction is seen in protective functions against biotic as well as abiotic stresses in the bacterial microbiome [15, 28]. Various further roles were assigned to prominent members of the symbiosis; a holistic picture remains to be generated. In our work on the lichen-associated microbial communities, we focused on the lung lichen *Lobaria pulmonaria* (L.) Hoffm., as a model organism. The leaf-like morphology of this lichen comprises a fungus that encloses a layer of green algae and intermingled clusters of cyanobacteria (so-called internal cephalodia, functional analogues of legume rhizobial nodules; [19]). This complex microenvironment makes this lichen an ideal object for further explorations of complex host-microbe interplay. Especially in life forms that are experimentally difficult, current omics approaches provide ideal tools for studies of functional diversity. These approaches yield also comprehensive baseline information for future experimental studies. For instance, a combined omics approach delivered important information on nitrogen fixation strategies employed by cyanobacteria within the lichen symbiosis [34].

So far, the application of meta-omics and amplicon sequencing facilitated the identification of the highly diversified bacterial microbiota of lichens, which seem commonly dominated by *Alphaproteobacteria* [3, 28, 31–33, 62]. We hypothesize that the lichen microbiota is composed of functional guilds, which fulfill distinct roles in the holobiome. By a first in-depth analysis, focusing on the predominant group of *Rhizobiales*, we detected signatures of nitrogen fixation, as well as synthetic potential of phytohormone and vitamin production [22]*.* Within the same taxonomic group, a so far unknown lineage of lichen-associated *Rhizobiales* was identified in a preceding study [31]. This major group represents potential “feeders” in the holobiome, while other smaller groups, e.g., the genera *Burkholderia*, *Paenibacillus*, and *Pseudomonas*, were identified as “protectors” [15, 16]. So far, little attention was paid to other bacterial lineages. We nevertheless hypothesize that besides their unrecovered diversity, such lineages could also contribute functionally to the adaptability and versatility of the symbiosis. Five bacterial orders with high occurrence in available meta-omics datasets were selected for the present study. These groups were repeatedly found in lichen microbiomes during the past years but remained unexplored in terms of their potential functions. Only recently, Aschenbrenner et al. [3] reported about the presence of *Spartobacteria* (*Verrucomicrobia*) in lichens, a poorly investigated group earlier found to be abundant in soil and aquatic environments [9, 37, 71]. New data reinforced the hypothesis that specific members of *Chthoniobacterales* (*Spartobacteria*) and *Sphingobacteriales* (*Bacteroidetes*) could potentially be more important colonizers of *L. pulmonaria* [4]. A major obstacle in studying the as yet uncultivable *Chthoniobacteria* was their absence in former releases of public sequence databases due to their rather low occurrence in terrestrial habitats and thus low presence in most environmental samples. The ecological significance of this and other abundant groups (*Sphingomonadales*, *Rhodospirillales*, *Myxococcales*, and *Sphingobacteriales*) will be assessed here by comparative analysis of a newly obtained metatranscriptomics dataset with metagenomic, metaproteomic, and 16S ribosomal RNA (rRNA) gene sequencing amplicon data.

## Methods

### Sampling of *L. pulmonaria*

Lichen thalli of *L. pulmonaria* were sampled in the Austrian Alps (Johnsbach, N 47° 32′ 35″, E 14° 37′ 38″; 1175 m above sea level) from a rich population on mountain maple bark (*Acer pseudoplatanus*) on 28 June 2014. Samplings were conducted in the late morning hours, when thalli were humidified (rather than dry) to ensure sufficient metabolic activity for transcriptome analysis. The samples were collected with sterile tweezers, cleaned from macroscopic contaminations (e.g., moss, bark, and insects), and immediately transferred into RNAlater®Stabilization solution (Ambion, Life Technologies, Germany) and stored at −20 °C until further processing.

### Meta-omics-based evaluations of bacterial community structures and functioning

16S rRNA gene amplicon and metagenomic datasets of the *Lobaria*-associated bacterial communities were retrieved from already published studies for comparative analyses with the two newly generated metatranscriptomic datasets. The available datasets were utilized for complementing analyses of the lichen microbiome [4, 28]. All datasets employed in this study were obtained with *L. pulmonaria* samples from the same sampling site. The 16S rRNA gene amplicon dataset consists of 24 barcoded samples while the utilized metagenome is based on one composite sample. Additional information about the different omics studies are listed in Additional file 1: Table S1.

### Metatranscriptome—sample preparation and sequencing

TRIZOL plus RNA purification kit was used to isolate total RNA according to the manufacturer’s protocol. The lichen tissue was homogenized with a FastPrep®-24 Instrument and FastPrep™ Lysing Matrix E (MP Biomedicals, Germany) for 3 × 30 s at 6.0 m/s with 1 min cooling on ice in between. Total RNA was purified with RNeasy mini kit (Qiagen, Germany), and RNA integrity was measured with Agilent 2100 Bioanalyzer (Agilent Technologies). To enrich mRNA for functional analysis, eukaryotic and prokaryotic ribosomal RNA was depleted (according to [44, 67]). For this purpose, rRNA probes specific for the eukaryotic and prokaryotic SSU and LSU rRNA regions were designed based on corresponding metagenomic DNA sequences. Small and large subunits of the rRNA gene fragments were amplified with specific primer sets (see Additional file 1: Table S2). Purified PCR products (Wizard ® SV Gel and PCR Clean-Up System, Promega, Germany) were used as DNA templates for the preparation of biotinylated antisense rRNA probes via in vitro transcription according to the manufacturer’s protocol (MEGAscript T7 kit, Ambion, Life Technologies, Germany). Finally, rRNA was subtracted from total RNA with streptavidin-coated magnetic beads after hybridization with the biotinylated anti-sense rRNA probes. Quality-checked RNA of three separately processed lichen thalli was pooled equimolar. Strand-specific complementary DNA (cDNA) library preparation of total and depleted RNA and Illumina HiSeq 2500 paired-end sequencing was performed by GATC Biotech AG (Konstanz, Germany).

### Taxonomic analysis

Downstream sequence analysis for taxonomical assignments was done using QIIME 1.9.0 [12]. The 16S rRNA gene fragment sequences were filtered from the genomic and transcriptomic datasets (deposited and publicly available on the metagenomics analysis server MG-RAST; IDs mgm4583748.3, mgm4745782.3) with SortMeRNA [42] based on the integrated bacterial 16S rRNA database (SILVA SSU Ref NR v.119; [50]). These sequences as well as the 16S rRNA gene amplicon sequences were clustered at 97% similarity based on the “pick_closed_reference_OTUs.py” script. The SILVA database (release 119; [57]) was used as reference sequence set for taxonomical assignment [56]. Mitochondrial and chloroplast sequences were removed from datasets with the implemented QIIME “filter_taxa_from_otu_table.py” script to omit non-bacterial reads. Each dataset was normalized to 5720 sequences, which equals the lowest sequence number in the limiting dataset. Taxonomic composition for each omic approach was visualized as Krona charts [52].

### Functional analysis

Functional analysis of the lichen-associated bacterial communities was performed with MG-RAST based on metagenomic and metatranscriptomic data. Sequences were compared to GenBank using a maximum *e* value of 1e−5 and a minimum identity cutoff of 70% [8]. All reads assigned to the orders *Sphingobacteriales* (*Bacteroidetes*), *Chthoniobacterales* (*Verrucomicrobia*), *Myxococcales*, *Sphingomonadales*, and *Rhodospirillales* (*Proteobacteria*) were extracted for further analysis. The short DNA/cDNA reads were aligned to the protein reference database NCBI-NR (version 05/2015) using DIAMOND (version 0.7.9; [10]). Functional assignment was performed with MEGAN5 [36] based on SEED classification [53]. The abundances of function-assigned sequences of the specified taxonomic orders were subsampled (1000 times randomly subsampled; default settings in MEGAN5) for comparison.

### Database search and metaproteome data analysis

The raw files were converted to mgf files by the Proteome Discoverer software (Thermo Scientific V1.3) and searched with the Mascot search engine (version 2.2.04, Matrix Science Inc.) with the following parameters: parent mass tolerance 10 ppm, fragment mass tolerance 0.5 Da, maximum missed cleavages 2, charge state 1+, and oxidation of methionine as variable modification. In order to improve the identification of high-confidence peptide sequence matches, a two-step database search was performed, similar to an approach described previously [38]. To this end, mass spectra were searched in a primary step against the NCBInr protein database (version 2014.06.25, 44,828,108 entries). Hits from the primary search were filtered according to the following parameters: 80% protein probability, 95% peptide probability, and a minimum of one uniquely identified peptide). Subsequently, protein identifiers fulfilling the above-described criteria were extracted. Based on these identifiers, a subset target-decoy database (34,344 entries) was constructed using an in-house script for local data deposition (“database creator”). Results from the second database search were filtered applying more stringent parameters (99% protein probability, 99% peptide probability, and a minimum of one uniquely identified peptide,) including a replicate filter, i.e., a protein had to be identified in two out of three technical replicates. Functional classification and taxonomic assignment of the protein sequences have been accomplished by the in-house developed metaproteome analyses pipeline “Prophane 2.0” (http://www.prophane.de, [62]). Relative protein quantification was based on normalized spectral abundance factor (NSAF) values [72], only considering spectral counts that have been uniquely identified for a specific protein.

### DNA isolation

Total DNA of each sample was extracted using the MoBioPowerSoil® DNA Isolation Kit (Carlsbad, USA) according to the manufacturer’s protocol with modifications from Aschenbrenner et al. [3]. Briefly, the samples were ribolyzed three times for 30 s at 5.5 m × s^−1^ and kept 5 min on ice in between. DNA from control strains (*Escherichia coli* *, Staphylococcus aureus, Pseudomonas aeruginosa*) was extracted following an ethanol precipitation protocol.

### Probe design, evaluation, and *Verrucomicrobia*-specific SSCP

Single-strand conformation polymorphism (SSCP) experiments were employed to validate the presence of *Verrucomicrobia* populations on lichens with a classic, molecular method. To target *Verrucomicrobia*, the oligonucleotide primers VMB537f and VMB1295r were initially used as in previous studies [51]. However, the specificity and coverage of the primers was not suitable for our approach (30% *Verrucomicrobia*, 17% *Spartobacteria*) after in silico tests against the Arb-Silva database using the TestPrime tool (http://www.arb-silva.de/search/testprime/). We therefore designed a new primer set. The forward primer (Verruco f) was designed based on the *Verrucomicrobia* FISH probe (EUB338III; 5′-GCTGCCACCCGTAGGTGT-3′; [20]). The annealing position for the forward primer is located at the upstream 330-bp position. The reverse primer (Sparto r; 5’-CCTTCGCCACTGGTCTTC-3′) was designed based on the *Spartobacteria* FISH probe [20]. The annealing position is located at about 680 bp upstream yielding in 350-bp fragments. According to TestPrime, 0.84% of all bacteria are amplified using these two primers Verruco f and 806r. This fraction includes 84% of the phylum *Verrucomicrobia* within 89% of the class *Spartobacteria* respectively *Chthoniobacterales*.

To assess the specificity of our primers, we produced *Verrucombicrobia*-specific fingerprints with various *Lobaria* samples (Additional file 1: Fig. S1). No band patterns were retrieved with control strains (*E. coli, S. aureus, P. aeruginosa)*, which represented the closest probeBase mismatches. Selected bands were sequenced and taxonomies were assigned by blast searches against the NCBI 16S ribosomal RNA sequence database. Using the novel primer approach, no additional taxa beyond *Verrucomicrobia* were detected within the selected sequencing range (Additional file 1: Fig. S2).

SSCP analysis of the total community DNA of four different samples, the type strain, and internal controls was carried out according to Schwieger and Tebbe [63] and Bassam et al. [31]. The PCR was performed using a total volume of 60 μl containing 12 μl of Taq&Go (QBiogen), 6 μl of the purified DNA, 1.5 μl of each primer (10 μM), and 39 μl of ultrapure water (95 °C, 5 min; 30 cycles of 95 °C, 20 s; 56.8 °C, 15 s; 72 °C, 30 s; final elongation at 72 °C, 10 min). Control strains (closest database mismatches according to probeBase; http://probebase.csb.univie.ac.at; *E. coli, S. aureus, P. aeruginosa*) were also amplified. The PCR products were purified using Wizard® SV Gel and PCR Clean-Up System (Promega, Madison, USA), prior to λ-exonuclease digestion and DNA single-strand folding [47]. Polyacrylamide gel electrophoresis was carried out on a TGGE platform (Biometra, Göttingen, Germany) at 26 °C and 400 V for 26 h using 8% (wt vol^−1^) acrylamide gel.

Since all sequences of the verrucomicrobial class *Spartobacteria* were assigned to *Chthoniobacterales*, we checked for specific FISH (fluorescent in situ hybridization) probes for the order *Chthoniobacterales* in probeBase (http://probebase.csb.univie.ac.at). As so order-specific probe exists, we used the probe SPA714 to detect specifically bacteria within Spartobacteria. However, this probe only targets about 66% (2553 out of 3874 hits) of all *Chthoniobacterales* sequences according to RDP probe match. Additionally, we evaluated the FISH probe EUB338III, which is supposed to specifically target the order *Verrucomicrobiales* (class *Verrucomicrobiae*) besides the non-target taxon *Chloroflexi*. Sequence alignments to the Probematch database [18] revealed that this probe does not only match to *Verrucomicrobiae* (6930/7266) but also covers the verrucomicrobial classes Subdivision 3 (3064/3647), *Opitutae* (4223/4460), and *Spartobacteria* (4441/4734). According to our data, about 89% (mean) of the verrucomicrobial sequences were assigned to the class *Spartobacteria*, followed by *Opitutae* (7%). Hence, this FISH probe is also suitable to detect Spartobacteria, and in a wider sense, *Chthoniobacterales* in the case of *L. pulmonaria-*associated bacterial community.

### FISH/CLSM

Fluorescence in situ hybridization (FISH) was performed to specifically visualize the colonization pattern of *Chthoniobacterales* (class *Spartobacteria*, phylum *Verrucomicrobia*) among other eubacteria. *L. pulmonaria* thalli were fixed with 4% paraformaldehyde/phosphate-buffered saline (PBS) with a ratio of 3:1 at 4 °C for at least 4 h. Prior to in situ hybridization according to Cardinale et al. [13], thallus cross-sections were prepared. For the detection of *Spartobacteria* as well as *Verrucomicrobia*, the Cy5-labled probes SPA714 (42 °C, 35% formamide; [2]) and EUB338III (42 °C, 15% formamide) were used. Other eubacteria were detected with an equimolar mixture of the Cy3-labeled probes EUB338, EUB338II, and EUB338III (42 °C, 15% formamide; [1, 20]). Additionally, NONEUB probes [69] labeled with the respective fluorochromes were used as negative controls. Details about oligonucleotide probes are available on probeBase [48]. To suppress photobleaching of the fluorescently labeled probes, SlowFade Diamond antifade reagent (Molecular Probes, Eugene, USA) was used. Targeted *Chthoniobacterales* and all other eubacteria were visualized with a Leica TCS SPE confocal laser-scanning microscope (Leica Microsystems, Mannheim, Germany) and confocal stacks were processed with Imaris 7.3 (Bitplane, Zurich, Switzerland).

## Results

### Comparative composition of the lichen microbiome

**Fig. 1:**
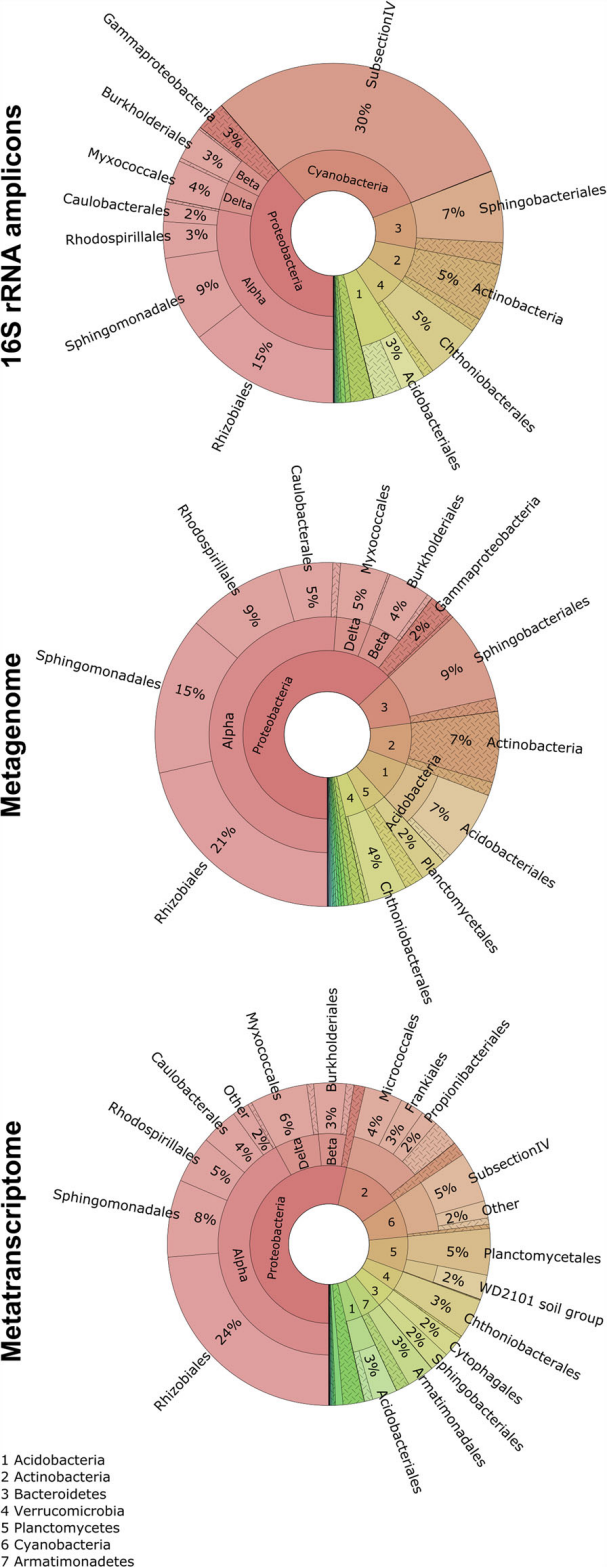
Bacterial community structures of the Lobaria-associated microbiome are visualized in a mulit-level Krona chart from phylum to order level (starting at the *inner circle*) for each omic approach (16S rNA amplicons, metagenome, metatranscriptome). Taxonomic information is based on the 16S rRNA gene fragment sequence analysis performed with QIIME

**Fig. 2:**
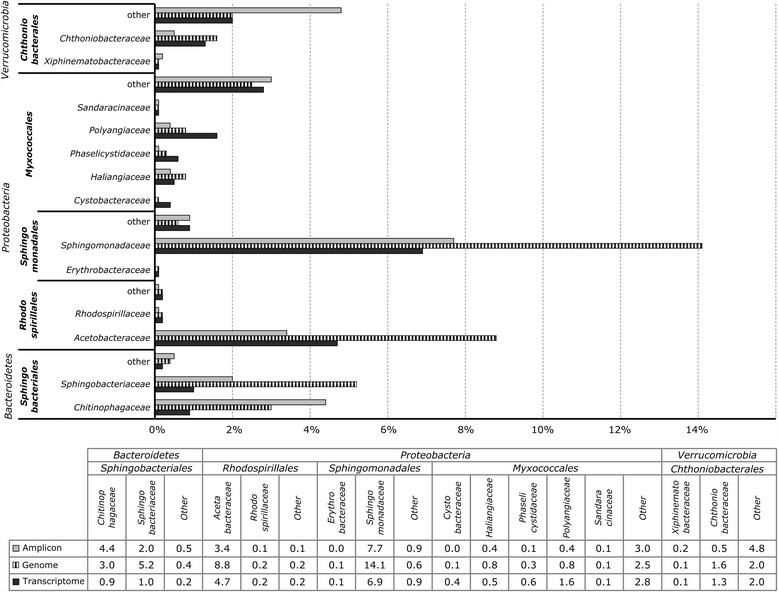
Relative sequence abundances of families within the highly abundant orders *Sphingobacteriales*, *Rhodospirillales*, *Sphingomonadales*, *Myxococcales*, and *Chthoniobacterales* according to the three omic approaches (16S rRNA amplicon, metagenome, and metatranscriptome). Unassigned or unclassified families were grouped into “others”

The general bacterial community structure showed certain analogies at phylum, class, and order levels across the analyzed datasets irrespective of approach (16S rRNA gene amplicon libraries, metagenome, metaproteome) and sample size (Fig. 1; Additional file 1: Table S1). Composition varied, however, at finer taxonomic resolution among the data sets with only 5.4% of the total OTUs being present in all datasets (Additional file 1: Fig. S3). The highest overlap in identical OTUs (16.8%) was found in the metagenome and metatranscriptome dataset overlay. Moreover, differences in the relative occurrence of distinct taxonomic lineages were evident. In particular, the abundance of *Cyanobacteria* showed remarkably high differences. In the amplicon dataset, *Cyanobacteria* comprised almost 30%, whereas they comprised less than 1% of the total reads in the metagenome, and 8% of the metabolically active community in the metatransciptome. In addition to the *Rhizobiales* and *Cyanobacteria* [22, 62], taxonomic analysis revealed five highly abundant orders within the bacterial community: *Sphingomonadales* (10.2 ± 3.3%; mean values and SD of the utilized omics and amplicon studies are provided), *Rhodospirillales* (5.8% ± 2.5%), *Myxococcales* (4.7 ± 0.8%, *Proteobacteria*), *Chthoniobacterales* (4.3 ± 0.7%, *Verrucomicrobia*), and *Sphingobacteriales* (5.8 ± 2.8%, *Bacteroidetes*). The composition and relative abundances of these orders at family level were visualized in Fig. 2. Within the order *Sphingobacteriales*, two main families were identified: *Sphingobacteriaceae* and *Chitinophagaceae*. The *Chitinophagaceae* were more abundant in the amplicon dataset when compared with the metagenome, whereas *Sphingobacteriaceae* were less abundant in the former dataset. *Chthoniobacterales* was represented by the families *Chthoniobacteraceae* and *Xiphinematobacteraceae*. Moreover, groups within *Chthoniobacteraceae* accounted for a lower fraction in the amplicon dataset than in the corresponding meta-omics data. Highly abundant families identified within the alphaproteobacterial orders *Sphingomonadales* were represented by *Sphingomonadaceae* and *Erythrobacteraceae*, as well as *Acetobacteraceae* and *Rhodospirillaceae* in *Rhodospirillales.* Both *Sphingomonadaceae* and *Acetobacteraceae* were a minor fraction in the amplicon data set in comparison with the metagenome data. The order *Myxococcales* was represented by various distinct families, whereas *Polyangiaceae*, *Phaselicystidaceae*, and *Cystobacteraceae* had a higher relative abundance in the metatranscriptome than in the other two meta-omics approaches. Other detected families within this order were *Haliangiaceae* and *Sandaracinaceae*.

### Functional contribution of abundant bacterial community members to the lichen symbiosis

**Fig. 3:**
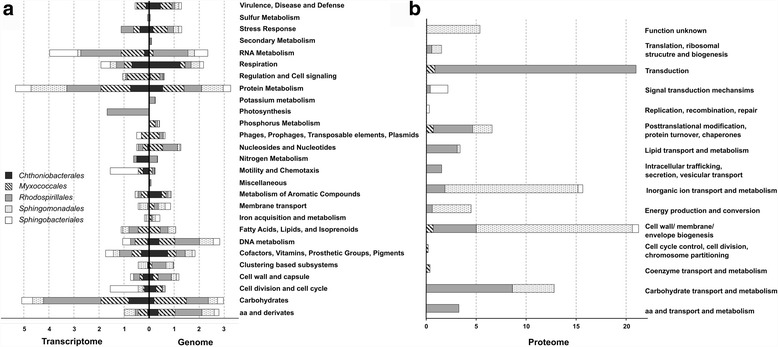
Functional assignments to selected orders within the Lobaria-associated microbiome based on the metagenome, transcriptome, and proteome. Functional information of *Chthoniobacterales*, *Myxococcales*, *Rhodospirillales*, *Sphingomonadales*, and *Sphingobacteriales* based on the transcriptome and genome was obtained by blastx assignment, followed by processing with MEGAN including SEED classification (**a**). The COG database was utilized for functional classification of the proteome data (**b**)

Together with metagenomic and metaprotemic data, the transcriptome information corroborated the functional potential on lichen-bacteria and provided expression data for the bacterial orders *Sphingomonadales*, *Rhodospirillales*, *Myxococcales*, *Chthoniobacterales*, and *Sphingobacteriales*. Functional assignments for each bacterial group based on SEED and COG classification are visualized in Fig. 3. During the analysis, we primarily focused on functions with a direct implication for the symbiosis or such that modulate interactions with the adjacent environment.

#### Provision of micronutrients (inkl. N, S, P, K, Fe metabolism)

In the metagenome, genes attributed to potassium and nitrogen metabolism were both predominantly (96%) assigned to the alphaproteobacterial order *Rhodospirillales* and to a minor extent to *Sphingobacteriales* (potassium metabolism) and *Sphingomonadales* (nitrogen metabolism). *Rhodospirillales* contributed at a high proportion (94 and 96%) to the assimilation of ammonia and potassium uptake and transport, respectively. There were also functional assignments to nitrate and nitrite ammonification and the production of nitric oxides. Sulfur and iron metabolism was predominately represented by *Sphingobacteriales*. Regarding iron metabolism, not only all *Sphingobacteriales*-specific reads contributed to the TonB-dependent receptor of Gram-negative bacteria but also all three proteobacterial taxa were involved in this iron acquisition strategy. Other identified iron transport mechanisms were systems based on siderophores or hemin. Functional genes within the phosphate metabolism were mainly assigned to *Proteobacteria*, especially *Myxococcales*. In contrast, nitrogen metabolism (ammonia assimilation) was mainly detected for *Chthoniobacterales* and only to a minor extent for *Rhodospirillales* in the metatranscriptome dataset. Also expressed genes related to both, iron acquisition and metabolism, were only detected for *Sphingobacteriales* and *Sphingomonadales* (including hemin transport system and a TonB-dependent receptor). With respect to iron transport, an outer membrane receptor protein detected in the metaproteome was also assigned to *Sphingomonadales*.

#### Aromatic compounds metabolism

All examined taxa were involved in the metabolism of aromatic compounds according to the analyzed metagenome. The majority of corresponding functions was assigned to various degradation mechanisms. *Chthoniobacterales* was the primary contributor to the degradation of n-phenylalkanoic acid. *Alphaproteobacteria* were involved in the degradation of phenylpropanoid, xylenols, and cresols (mainly by *Rhodospirillales*) and chloroaromatic compounds (*Myxococcales*). Especially, *Proteobacteria* were identified to potentially synthesize the plant hormone auxin. *Rhodospirillales* was also found to be involved in phenazine biosynthesis. In the metatranscriptome data, phenazine and auxin production were not present for any of the investigated groups. Instead, degradation and transport mechanisms of various compounds were found, such as biphenyl, carbazol, and benzoate (*Sphingomonadales*) as well as gentisate and salicylate (*Rhodospirillales*, *Myxococcales*).

#### Cofactors, vitamins, prosthetic groups, and pigments

*Chthoniobacterales* encoded genes involved in the syntheses of the vitamins riboflavin and biotin, whereas, genes for the thiamin, pyridoxine, and folate syntheses were predominantly assigned to *Proteobacteria*. However, transcriptomic data revealed biotin and folate synthesized by *Chthoniobacterales*. The vitamins riboflavin, pyridoxine, and thiamin were mainly produced by *Rhodospirillales*. Moreover, *Sphingomonadales* expressed the cobalamin-adenosyltransferase PduO (EC 2.5.1.17), which is involved in the biosynthesis of vitamin B12. Quinone cofactors were found to be synthesized by *Sphingobacteriales* (menaquinone) and *Myxococcales*, whereas the latter one expressed 4-hydroxyphenylpyruvate dioxygenase (EC 1.13.11.27) which is involved in the biosynthesis of plastoquinone and tocopherol.

#### Stress response

Function-related genes within the category stress response were mainly assigned to oxidative stress and heat shock. According to the metagenome, the latter one was predominantly represented by *Myxococcales* and *Sphingobacteriales* encoding for the chaperones DnaK and GroEL. DnaK was also found to be expressed by *Myxococcales* and GroEL by *Rhodospirillales* and *Sphingomonadales* according to transcriptomic and proteomic data, respectively. Additionally, various sigma factors could be detected either based on RNA or protein sequences: RpoH (*Rhodospirillales*) for heat shock, RpoN (*Sphingobacteriales*) for nitrogen-limitation, and a homolog of the exocytoplasmic heat stress sigma factor RpoE (*Myxococcales*). Notably, all of the observed taxa encoded to a certain extent defense mechanisms against oxidative stress. In particular, *Chthoniobacterales* encoded for a redox-sensitive transcription regulator and *Rhodospirillales* for rubrerythrin, which is involved in oxidative stress tolerance in anaerobic bacteria. All *Proteobacteria* facilitated glutathione-dependent protection against ROS-induced (reactive oxygen species) oxidative damage. Although the catalase EC 1.11.1.6 was found to be encoded in all observed *Proteobacteria*, transcripts could be only detected for *Myxococcales*. According to transcriptomic data, *Chthoniobacterales*, *Myxococcales,* and *Rhodospirillales* were involved in the syntheses of glutaredoxin-related proteins, glutaredoxin, and rubrerythrin and glutathione for non-redox reactions. Moreover, hits for iron/manganese superoxide-dismutases were found in the proteome. Resistance mechanisms against acid stress (arginine decarboxylase EC 4.1.1.19) were additionally detected for *Myxococcales*.

#### Virulence, disease, and defense

Resistance to antibiotics and toxic compounds was predominantly shown by *Chthoniobacterales* and *Myxococcales*. Based on the metagenome, the latter one exhibited resistances to the metals arsenic and zinc. In addition, a response regulator of zinc sigma-54-dependent two-component system could be detected in the transcriptomic data. Resistance to cobalt and cadmium was identified for *Sphingomonadales* and *Sphingobacteriales*. Regarding antibiotic resistances, all taxa encoded for protection mechanisms against fluoroquinolones, corresponding sequences in the transcriptome could be also detected for *Sphingobacteriales*, *Myxococcales* and *Chthoniobacterales*. The latter one exhibited additionally resistance to acriflavin. Beta-lactamases were detected especially for *Alphaproteobacteria* and *Sphingobacteriales*, whereas multidrug resistance, e.g., via efflux pumps, were found for all taxa, except for *Myxococcales*. Proteomic data revealed no hits for functions related to resistance mechanisms.

#### Membrane transport

Various types of transport systems were found in the metagenomic dataset, such as protein secretion system types II, III, and IV. Secretion system type IV was found in all observed taxa except for *Chthoniobacterales*. Additionally, *Rhodospirillales* encoded for the sec-independent twin-arginine translocation pathway to transport folded proteins as well as manganese, zinc, nickel, and cobalt. Functional assignments to Ton- and Tol-dependent transport systems were found for all observed taxa. Metatranscriptomic data displayed the expression of Ton and Tol transport systems for *Sphingomonadales* and *Sphingobacteriales*, as well as hits for parts of the type IV secretion system in *Rhodospirillales*. TonB-dependent receptor proteins (*Sphingomonadales* and *Sphingobacteriales*) and protein export chaperone SecB (*Rhodospirillales*) were also found in the metaproteome.

#### Motility and chemotaxis

*Proteobacteria*, and *Myxococcales* especially, were involved in chemotaxis encoding the proteins methyltransferase CheR (EC 2.1.1.80) and the methylesterase CheB (EC 3.1.1.61). According to the metatranscriptome, only the signal transduction histidine kinase CheA was detected for *Myxococcales*. *Rhodospirillales* predominantly encoded for flagellar motility. Contrarily, in the metatranscriptome, the main contributor to flagellar motility was *Sphingobacteriales*, followed by *Chthoniobacterales* and *Myxococcales*.

#### Occurrence of phages, prophages, transposable elements, and plasmids

Functional assignments within this category were only found for *Sphingomonadales* and *Sphingobacteriales*, including transposable elements like Tn552 encoding for a beta-lactamase or the staphylococcal phi-Mu50B-like prophages. In the metatranscriptome analysis, transcripts for the staphylococcal pathogenicity island SaPI were found for *Sphingobacteriales* and *Myxococcales*.

### *Verrucomicrobia*-specific microbial fingerprints (PCR-SSCP) and sequencing revealed several lineages of *Chthoniobacterales*

PCR amplicons retrieved using *Verrucomicrobia*-specific primer were separated by SSCP (single-strand conformation polymorphism). The phylogenetic analysis of sequences obtained from excised products revealed three lineages in *Chthoniobacteriales*. One of these is related to *Chthoniobacter*, a second represents relatives of *Udaeobacter*, and a third lineage has no relatives among known genera in the order.

### Visualization of *Chthoniobacterales* within the lichen symbiosis

**Fig. 4:**
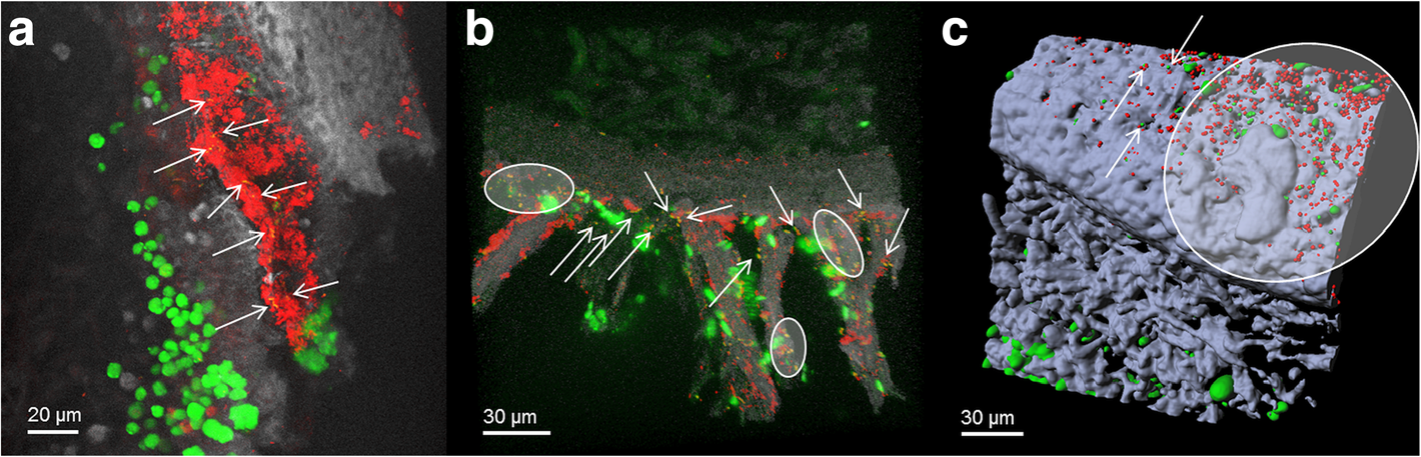
Colonization patterns of *Spartobacteria* (**a**, **b**) and *Verrucomicrobia* (**c**) on the lichen thallus of *L. pulmonaria* stained by fluorescence in situ hybridization (FISH). *Green*: algae *Dictyochloropsis reticulata*; *gray* or *blue/purple*: lichenized fungus *L. pulmonaria*; *yellow*: *Spartobacteria* or *Verrucomicrobia*; *red*, other eubacteria. **a**, **b** Volume rendering of confocal stacks. **c** Three-dimensional model reconstruction visualized as isosurface and spheres. *Arrows* or *circles* indicate single colonies or areas with small clusters of *Spartobacteria* or *Verrucomicrobia* within unspecific-labeled bacterial aggregates

The microscopic localization of *Chthoniobacterales* was utilized to explore colonization patterns and potential accumulations of these previously underexplored colonizers. Fluorescence in situ hybridization with *Spartobacteria*-specific probes revealed consistent colonization of both the upper and the lower cortex of *L. pulmonaria* (Fig. 4a, b). Volume rendering of the confocal stacks clearly visualized a general tendency of *Spartobacteria* to be dispersed on the lichen as single small colonies between biofilm-like structures of other eubacteria (which were not specified further). Although there were also small areas with higher cell densities (Fig. 4b), no larger colonies of *Spartobacteria* were detected. Micrographs with the *Verrucomicrobia-*specific probe confirmed these colonization patterns (Fig. 4c).

## Discussion

In the present study, we show how the addition of transcriptomic data to genomic and proteomic information contributes to the detection of so far unknown active bacterial players in the lichen symbiosis. This approach is backed up by their complementary microscopic FISH/CLSM visualization. Lichens have complex and functionally diverse microbiota. As some may serve in nutrition of the host while others provide protective functions, or growth regulating functions to the hosts, we may generally distinguish feeders and protectors. The obtained data indicates that members of the identified functional guild are found in both roles and primarily serve as probiotics and detoxifiers in the *Lobaria* holobiome. Similarly, Hodkinson et al. [34] employed a metatranscriptomics-based approach to explore detailed functioning of nitrogen fixation by lichen-symbiotic cyanobacteria.

**Fig. 5:**
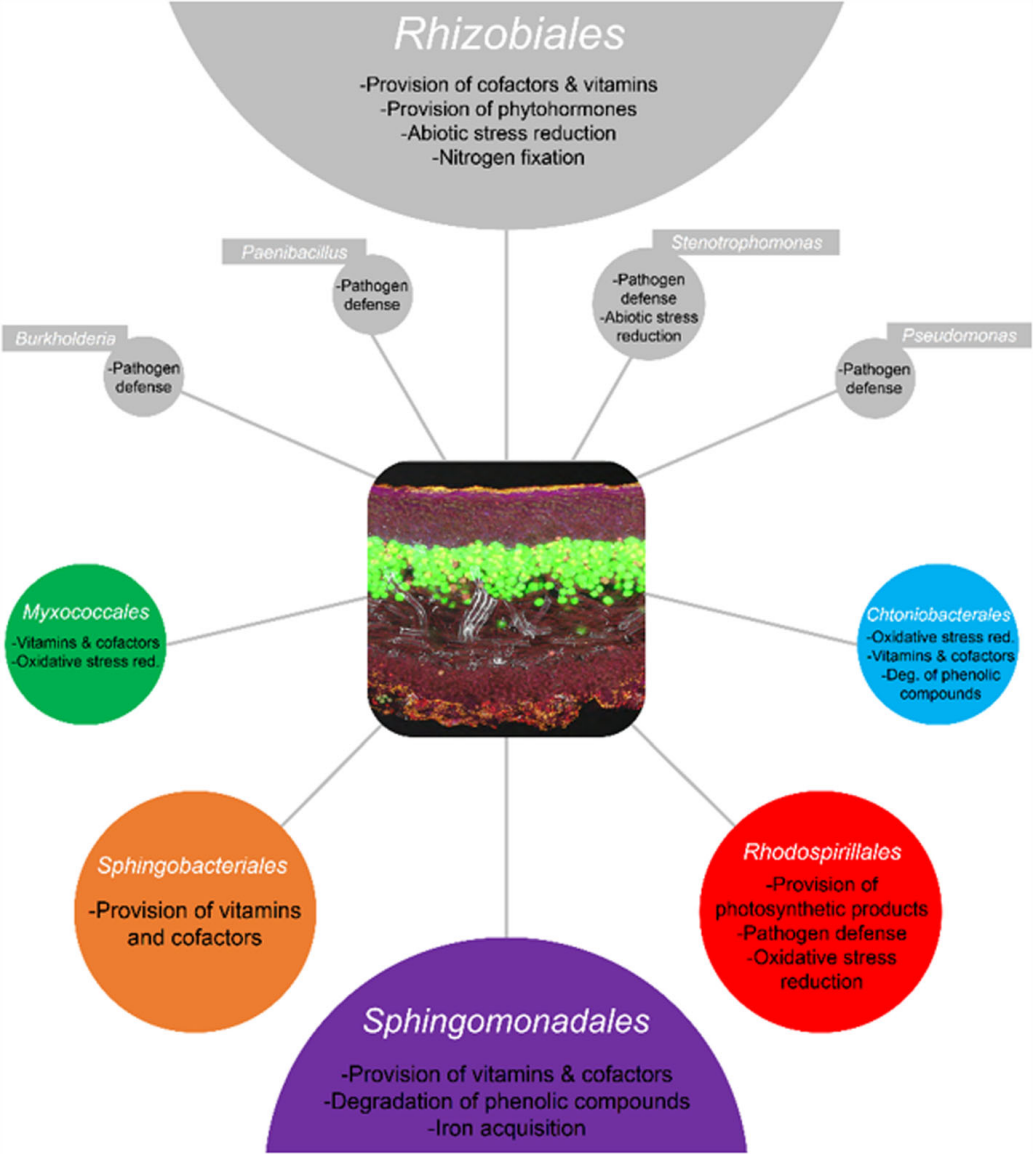
Functional assignments within the lichen symbiosis obtained with multi-omics technologies and corresponding analyses. Bacterial groups are shown on different taxonomic levels. *Colored bubbles* include results from the current study, while *gray bubbles* were adapted with data from Cernava et al. [15, 16], Erlacher et al. [22], and Grube et al. [28]. Bubble sizes correspond to the mean abundance of each group, such with a rectangular label field are present with <1% in the *Lobaria* microbiome

In the current study, specific functional contributions to the overall symbiosis were explored in five abundant bacterial orders, namely *Sphingomonadales*, *Rhodospirillales*, *Myxococcales* (all *Proteobacteria*), *Chthoniobacterales* (*Verrucomicrobia*), and *Sphingobacteriales* (*Bacteroidetes*) (Fig. 5). Both metagenomic and metatranscriptomic analyses revealed various strategies of associated bacteria to survive under stressful conditions. Lichens are known to produce extracellular reactive oxygen species (ROS) including superoxide, hydroxyl radicals, and hydrogen peroxide [6], which are produced at high rates after desiccation-rehydration events. According to Beckett et al. [7], extracellular ROS production might also help defending against bacterial and fungal pathogens in this lineage of lichenized fungi, which largely lacks crystals of potentially antimicrobial lichen compounds in the surface layers. In general, fast-growing bacterial pathogens are not known to degrade lichens and many bacterial associates rather represent stress-tolerant commensals or even beneficials, which contribute various functions to the lichen meta-organism [22, 28]. These bacteria require efficient mechanisms to withstand periodic desiccation/rehydration cycles with associated oxidative bursts. Metagenomic data support this hypothesis as corresponding mechanisms against oxidative stress, such as catalases and low-molecular-weight antioxidants (e.g., glutathione) are encoded across bacterial groups. These functions were found also for the active fraction in the metatranscriptome.

Oxidative stress conditions also induce the expression of various heat shock proteins [68]. In particular, the chaperones GroEL and DnaK, which were highly abundant in our data, can be induced by various oxygen species such as superoxide or hydrogen peroxide, respectively [23], or by the presence of toxic compounds like antibiotics, heavy metals, and aromatic compounds [68]. Several functions, which protect against such toxic compounds, were also reflected in our data, e.g., those contributing to resistances to the toxic metals arsenic, cadmium, cobalt, or zinc. In addition, resistance mechanisms for antibiotics such as fluoroquinolones were also found like beta-lactamases and efflux pumps for multidrug resistances. As secondary metabolites (including depsides, depsidones, and dibenzofurans) produced by the lichenized fungus have antimicrobial activities to defend the host against fungal and bacterial pathogens [43], lichen- and plant-associated bacteria likely developed different defense mechanisms against these specific aromatic compounds. Our data confirmed the presence of potential functions to degrade these metabolites such as phenylpropanoid, which is the carbon skeleton of a wide range of polyphenols. In contrast, there were also hints for the production of phenolic compounds by bacteria themselves. Especially, *Rhodospirillales* was found to be involved in the production of phenazines, which are known to inhibit the bacterial and fungal growth [49] thereby increasing its own competitiveness and ecological fitness [54]. This could also have a positive effect for the lichen itself as these broad-specificity antibiotics might control fast-growing bacterial and fungal pathogens.

According to our omics datasets, the observed taxa encode and express various transport machineries such as Ton- and Tol-dependent transport systems as well as different secretion systems. In this context, the type IV secretion system (T4SS) is also interesting, as it is not only involved in the DNA uptake from or the release to the surrounding environment or in the conjugal DNA transfer but also in the translocation of effector molecules to eukaryotic target cells [21]. These trans-kingdom transfers include fungi, plants, and mammalian cells [11, 14, 70]. We hypothesize that T4SS might be also used for inter-kingdom “cross-talking” between the lichen and members of the associated microbiome, analogous to the Rhizobia-plant interaction [26, 64].

In this work, we showed the metabolic contribution of the hitherto overlooked phylum *Verrucomicrobia*. After the discovery of *Chthoniobacter flavus* in soil [60], only a few studies so far focused on *Spartobacteria* as a bacterial class of *Verrucomicrobia* [39, 61]. According to genomic data, *Spartobacteria* have roles in the carbon cycle by in degradation of various complex carbohydrates (such as cellulose and xylan; [30]). With newly designed oligonucleotides, we were now able to visualize the presence of *Chthoniobacterales* for the first time in lichens. Transcriptomics data suggest their involvement in the metabolism of aromatic compounds (degradation of phenolic substances), production of various vitamins, and defense against antibiotics (fluoroquinolone) and oxidative stress. Interestingly, no information on this particular group was found in the metaproteome, which might be due to the lack of data in incomplete reference databases.

The new transcriptomic data of the lichen microbiome validate and corroborate the evidence for particular functions, but appropriate integration of different omics approaches nevertheless require further optimization of lab procedures and bioinformatic analyses. Variation may occur during sample preparation for individual omics approaches [66]. Primer selection has an impact on representation of taxonomic composition in amplicon sequencing [25, 41, 55], which may be further complicated due to gene copy number variations in the rRNA operons [40, 58]. Community composition in metatranscriptomic data is influenced by bacteria-specific differences of metabolic activity. Similar applies also to representation of the metaproteome, which is also sensitive to the preparation of the protein fraction. In contrast, the metagenome may also comprise a fraction of inactive or dead bacteria, which were recently visualized by Cernava et al. [17]. Since predominant members of the lichen microbiota remain fairly stable [27], we exclude that different sampling dates are of significant influence.

With the combination of different approaches, our goal is a better understanding of symbiotic interactions in the microbial system of lichens with their well-defined architecture, and their ability to grow and diversify in nutrient-deprived and stressful environments. Hays et al. [29] pointed out “symbioses provide a way to surpass the limitations of individual microbes.” With the recent findings, lichen symbioses are now viewed as extended symbiotic networks with further interacting bacterial partners, beside fungi and algae. We show that the functionally diverse communities of bacteria on the surfaces of the lichens provide additional benefits beside the algal partners. Our vision is to use the insights from this natural symbiotic system also as inspirations for novel biotechnological applications, which surpass the era of axenic culture.

## Conclusions

The present study allowed further advancements in the exploration of the lichen holobiome. Previously unexplored functional guilds of this unconventional model were captured with independent methods and further characterized in an integrative approach. It was shown that *Sphingomonadales*, *Rhodospirillales*, *Myxococcales*, *Chthoniobacterales,* and *Sphingobacteriales* account for consistent fractions of the *L. pulmonaria* microbiome irrespective of the utilized meta-omics tool. Deepening analyses provided insights into functional contributions of this bacterial consortium, which was then brought into an ecological context. By combining various meta-omic techniques, deficiencies of the particular methods were bypassed. Cost-efficient amplicon sequencing studies are often preferred when microbial community structures are explored in novel habitats. However, they deliver less comprehensive information compared to metagenomic and metatranscriptomic datasets. The combination of the advantages of various NGS methods provides generally a higher accuracy for a holistic study. Taken together, we successfully applied a combination of state-of-the-art tools for a deepening exploration of a complex multi-partner network.

## Additional file

Additional file 1: Table S1. Metadata and basic information related to the DNA/RNA-based omic approaches. **Table S2:** Primer sets for eukaryotic and prokaryotic rRNA gene fragment amplification. Bold: T7 promoter sequence; blue: enhancer sequence. **Table S3:** Taxonomical assignments. **Fig. S1:** Molecular fingerprints of 16S rRNA gene fragments from total DNA (Tot DNA) from the lichen, isolates from the lichen thalli (*L. pulmonaria*) and control strains (*E. coli, S. aureus, P. aeruginosa*). **Fig. S2:** Phylogenetic tree of bacteria-strains within the phylum *Verrucomicrobia*. The tree was constructed from an evolutionary distance matrix based on the neighbor-joining method [59]. The phylogenetic tree was constructed with 1000 seeds and 1000 bootstraps with the neighboring-joining method [59] using clustalX2 [45] and phylip [24]. **Fig. S3:** Common and unique OTUs among three omics approaches based on 16S rRNA gene fragment sequences with a genetic distance of 3%.
