# Supplementary material for: Deciphering functional diversification within the lichen microbiota by meta-omics
Source: Microbiome. 2017 Jul 19;5:82. doi: 10.1186/s40168-017-0303-5 (PMC5518139; doi:10.1186/s40168-017-0303-5)
Supplement: Additional file 1: Table S1. — Metadata and basic information related to the DNA/RNA-based omic approaches. Table S2: Primer sets for eukaryotic and prokaryotic rRNA gene fragment amplification. Bold: T7 promoter sequence; blue: enhancer sequence. Table S3: Taxonomical assignments. Fig. S1: Molecular fingerprints of 16S rRNA gene fragments from total DNA (Tot DNA) from the lichen, isolates from the lichen thalli (L. pulmonaria) and control strains (E. coli, S. aureus, P. aeruginosa). Fig. S2: Phylogenetic tree of bacteria-strains within the phylum Verrucomicrobia. The tree was constructed from an evolutionary distance matrix based on the neighbor-joining method [59]. The phylogenetic tree was constructed with 1000 seeds and 1000 bootstraps with the neighboring-joining method [59] using clustalX2 [45] and phylip [24]. Fig. S3: Common and unique OTUs among three omics approaches based on 16S rRNA gene fragment sequences with a genetic distance of 3%. [file 40168_2017_303_MOESM1_ESM.docx]

# Additional file 1

Table S1: Metadata and basic information related to the DNA/RNA-based omic-approaches

| **method** | **sampling site and date** | **sample preparation** | **sequencing platform** | **# of sequences (post QC)** | **mean sequence length (post QC)** | **public repository accession** | **first utilization of the data** |
| --- | --- | --- | --- | --- | --- | --- | --- |
| Metagenome | Johnsbach/Austria 12. October 2012 | Total DNA extraction from one composite biological sample consisting of 176.3 g *L. pulmonaria* thalli | Illumina HiSeq 2000 | 67,731,962 | 143 ± 29 bp | MG-RAST ID: mgm4530091.3 | Grube et al. (2015) |
| Metatranscriptome total RNA | Johnsbach/Austria 28. June 2014 | Total RNA isolation from three *L. pulmonaria* thallus fragments and subsequent equimolar pooling into one composite sample | Illumina HiSeq 2500 | 2,788,208 | 104 ± 19 bp | MG-RAST ID: mgm4583748.3 | this study |
| Metatranscriptome  Depleted RNA | Johnsbach/Austria 28. June 2014 | Total RNA isolation from three *L. pulmonaria* thallus fragments and subsequent equimolar pooling into one composite sample | Illumina Hiseq 2500 | 91,421,979 | 112 ± 27 bp | MG-RAST ID: mgm4745782.3 | this study |
| 16S rRNA gene amplicons | Johnsbach/Austria 17. October 2014 | Barcoding of 24 *L. pulmonaria* samples followed by amplicon sequencing | Illumina MiSeq | 415,693 | 290 ± 10 bp | NCBI SRA accession no.: PRJNA290145 | Aschenbrenner et al. (2017) |

Table S2: Primer sets for eukaryotic and prokaryotic rRNA gene fragment amplification. Bold: T7 promoter sequence; blue: enhancer sequence

| **Primer name** | **Sequence (5'-->3')** |
| --- | --- |
| **Eukaryotic SSU** | |
| NS1 | GTAGTCATATGCTTGTCTC |
| SR2_T7 | GCCAGTGAATTG**TAATACGACTCACTATAGG**CGGCCATGCACCACC |
| SR8R | GAACCAGGACTTTTACCTT |
| NS8_T7 | GCCAGTGAATTG**TAATACGACTCACTATAGG**TCCGCAGGTTCACCTACG |
| **Eukaryotic LSU** | |
| LR0R | ACCCGCTGAACTTAAGC |
| LR7_T7 | GCCAGTGAATTG**TAATACGACTCACTATAGG**TACTACCACCAAGATCT |
| LR7R | GCAGATCTTGGTGGTAG |
| LR14_T7 | GCCAGTGAATTG**TAATACGACTCACTATAGG**AGCCAAACTCCCCACCTG |
| **Prokaryotic SSU** | |
| 803R_T7 | GCCAGTGAATTG**TAATACGACTCACTATAGG**NCTACCTGGGTATCTAATCC |
| 347F | GGAGGCAGCAGTRRGGAAT |
| 1492R_T7 | GCCAGTGAATTG**TAATACGACTCACTATAGG**GACGGCTACCTTGTTACGACTT |
| **Prokaryotic LSU** | |
| 189F | GAASTGAAACATCTHAGTA |
| 2490R_T7 | GCCAGTGAATTG**TAATACGACTCACTATAGG**GCGACATCGAGGTGCCAAAC |
| 1075F | GTTGGCTTRGARGCAGC |
| 2241R_T7 | GCCAGTGAATTG**TAATACGACTCACTATAGG**GACCGCCCCAGTHAAACT |

Table S3: .Taxonomical assignments

| Sequence | **NCBI blastn, nr/nt, megablast** |  | **NCBI blastn, refseq_rna, megablast** |  | **NCBI blastn, 16S rrna, megablast** |  |
| --- | --- | --- | --- | --- | --- | --- |
| 1 | Chthoniobacter flavus strain Ellin428 | 100% | Chthoniobacter flavus Ellin428 | 100% | Chthoniobacter flavus Ellin428 | 100% |
| 2 | Chthoniobacter flavus strain Ellin428 | 100% | Chthoniobacter flavus Ellin428 | 100% | Chthoniobacter flavus Ellin428 | 100% |
| 3 | Verrucomicrobia bacterium SCGC AAA487-O09 | 93% | Chthoniobacter flavus Ellin428 | 89% | Chthoniobacter flavus Ellin428 | 89% |
| 4 | Verrucomicrobia bacterium SCGC AAA487-O09 | 90% | Chthoniobacter flavus Ellin428 | 88% | Chthoniobacter flavus Ellin428 | 88% |
| 5 | Verrucomicrobia bacterium SCGC AAA487-O09 | 90% | Chthoniobacter flavus Ellin428 | 89% | Chthoniobacter flavus Ellin428 | 89% |
| 6 | Verrucomicrobia bacterium SCGC AAA487-O09 | 93% | Chthoniobacter flavus Ellin428 | 90% | Chthoniobacter flavus Ellin428 | 90% |
| 7 | Verrucomicrobia bacterium SCGC AAA487-O09 | 95% | Chthoniobacter flavus Ellin428 | 90% | Chthoniobacter flavus Ellin428 | 90% |
| 8 | Verrucomicrobia bacterium WSF2-44 | 91% | Chthoniobacter flavus Ellin428 | 88% | Chthoniobacter flavus Ellin428 | 88% |
| 9 | Verrucomicrobia bacterium WSF2-44 | 96% | Chthoniobacter flavus Ellin428 | 90% | Chthoniobacter flavus Ellin428 | 90% |
|  |  |  |  |  |  |  |
|  | **LCA tax silva, SSU, 80% CT,** |  | **RDP (80% CT, 16S rrna training set 16)** |  | **Greengenes (arb-silva aligner, 80% CT)** |  |
| 1 | Chthoniobacter | 92% | Spartobacteria_genera_incertae_sedis | 100% | Chthoniobacter | 92% |
| 2 | Chthoniobacter | 93% | Spartobacteria_genera_incertae_sedis | 100% | Chthoniobacter | 93% |
| 3 | Chthoniobacterales | 90% | Spartobacteria_genera_incertae_sedis | 65% | Chthoniobacteraceae | 90% |
| 4 | Chthoniobacterales | 88% | Spartobacteria_genera_incertae_sedis | 84% | Chthoniobacteraceae | 88% |
| 5 | Chthoniobacterales | 89% | Spartobacteria_genera_incertae_sedis | 80% | Chthoniobacteraceae | 89% |
| 6 | Chthoniobacterales | 93% | Spartobacteria_genera_incertae_sedis | 87% | Chthoniobacteraceae | 93% |
| 7 | Chthoniobacterales | 94% | Spartobacteria_genera_incertae_sedis | 72% | Chthoniobacteraceae | 94% |
| 8 | DA101 (Chthoniobacterales) | 95% | Spartobacteria_genera_incertae_sedis | 100% | DA101 (Chthoniobacteraceae) | 95% |
| 9 | DA101 (Chthoniobacterales) | 94% | Spartobacteria_genera_incertae_sedis | 100% | DA101 (Chthoniobacteraceae) | 94% |


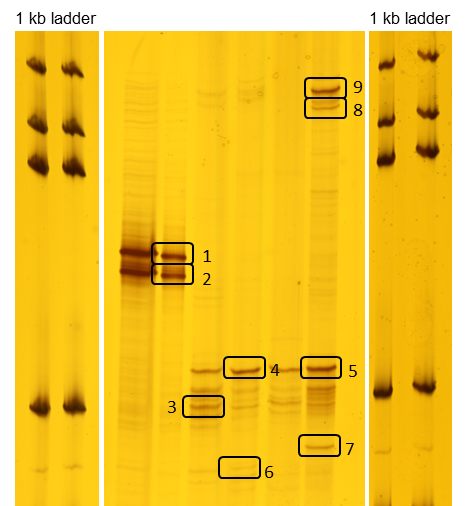


Figure S1: Molecular fingerprints of 16S rRNA gene fragments from total DNA (Tot DNA) from the lichen, isolates from the lichen thalli (*L. pulmonaria*) and control strains (*E. coli, S. aureus, P. aeruginosa*).

Figure S2: Phylogenetic tree of bacteria-strains within the phylum *Verrucomicrobia*. The tree was constructed from an evolutionary distance matrix based on the neighbor-joining method (Saitou and Nei, 1987). The phylogenetic tree was constructed with 1000 seeds and 1000 bootstraps with the neighboring-joining method (Saitou and Nei, 1987) using clustalX2 (Larkin *et al*., 2007) and phylip (Felsenstein, 1981).


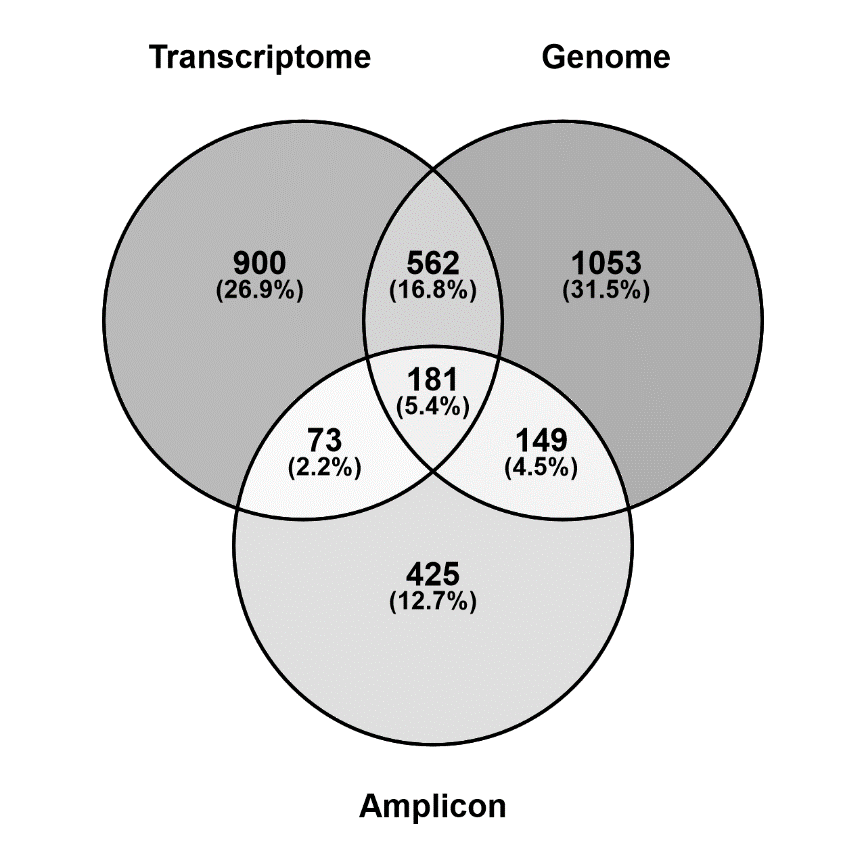


Figure S3: Common and unique OTUs among three omics-approaches based on 16S rRNA gene fragment sequences with a genetic distance of 3%.
